# Human resources for primary health care in sub-Saharan Africa: progress or stagnation?

**DOI:** 10.1186/s12960-015-0073-8

**Published:** 2015-09-10

**Authors:** Merlin L Willcox, Wim Peersman, Pierre Daou, Chiaka Diakité, Francis Bajunirwe, Vincent Mubangizi, Eman Hassan Mahmoud, Shabir Moosa, Nthabiseng Phaladze, Oathokwa Nkomazana, Mustafa Khogali, Drissa Diallo, Jan De Maeseneer, David Mant

**Affiliations:** Nuffield Department of Primary Care Health Sciences, University of Oxford, Oxford, UK; Department of Family Medicine and Primary Health Care, Ghent University, Ghent, Belgium; Department of Community Health, Mbarara University of Science and Technology, Mbarara, Uganda; Faculty of Medicine, Pharmacy and Dentistry, University of Bamako, Bamako, Mali; Department of Traditional Medicine, National Institute for Public Health Research, Bamako, Mali; Ahfad University for Women, Omdurman, Sudan; Department of Family Medicine, University of Witwatersrand, Johannesburg, South Africa; School of Nursing, University of Botswana, Gaborone, Botswana; School of Medicine, University of Botswana, Gaborone, Botswana

**Keywords:** Human resources for health, Primary health care, Review, Sudan, Mali, Uganda, Botswana, South Africa

## Abstract

**Background:**

The World Health Organization defines a “critical shortage” of health workers as being fewer than 2.28 health workers per 1000 population and failing to attain 80% coverage for deliveries by skilled birth attendants. We aimed to quantify the number of health workers in five African countries and the proportion of these currently working in primary health care facilities, to compare this to estimates of numbers needed and to assess how the situation has changed in recent years.

**Methods:**

This study is a review of published and unpublished “grey” literature on human resources for health in five disparate countries: Mali, Sudan, Uganda, Botswana and South Africa.

**Results:**

Health worker density has increased steadily since 2000 in South Africa and Botswana which already meet WHO targets but has not significantly increased since 2004 in Sudan, Mali and Uganda which have a critical shortage of health workers. In all five countries, a minority of doctors, nurses and midwives are working in primary health care, and shortages of qualified staff are greatest in rural areas. In Uganda, shortages are greater in primary health care settings than at higher levels. In Mali, few community health centres have a midwife or a doctor. Even South Africa has a shortage of doctors in primary health care in poorer districts. Although most countries recognize village health workers, traditional healers and traditional birth attendants, there are insufficient data on their numbers.

**Conclusion:**

There is an “inverse primary health care law” in the countries studied: staffing is inversely related to poverty and level of need, and health worker density is not increasing in the lowest income countries. Unless there is money to recruit and retain staff in these areas, training programmes will not improve health worker density because the trained staff will simply leave to work elsewhere. Information systems need to be improved in a way that informs policy on the health workforce. It may be possible to use existing resources more cost-effectively by involving skilled staff to supervise and support lower level health care workers who currently provide the front line of primary health care in most of Africa.

## Introduction

Primary health care is widely recognized as the most cost-effective strategy for delivering essential health interventions, for example, to reduce maternal and child mortality [1-3]. According to the Declaration of Alma-Ata, primary health care “addresses the main health problems in the community, providing promotive, preventive, curative and rehabilitative services accordingly…. [it] relies, at local and referral levels, on health workers, including physicians, nurses, midwives, auxiliaries and community workers as applicable, as well as traditional practitioners as needed, suitably trained socially and technically to work as a health team and to respond to the expressed health needs of the community” [4].

Reducing maternal and child mortality are key public health aims, embodied in the Millennium Development Goals [5]. Although there are good evidence-based interventions to achieve these goals [2], an important constraint in implementing them is the shortage of human resources, particularly in primary health care.

There is no agreed international standard for overall staffing of primary health care. In 2006, the World Health Organization defined countries as having a “critical shortage” of health workers if they had fewer than 2.28 doctors, nurses and midwives per 1000 population and if they failed to reach the target of 80% of deliveries being attended by a “skilled birth attendant”[6]. Fifty-seven countries met this definition, 36 of them in sub-Saharan Africa. However, these statistics do not differentiate between staffing in primary health care and in higher level health facilities.

This study was part of a larger project intended to address human resources for primary health care in sub-Saharan Africa. Countries were selected on two criteria: (1) links with participating EU countries (a prerequisite for the grant application) and (2) they exemplified a range of primary health care systems, geographic, socio-economic and political situations: Mali in West Africa, Sudan in Northern Africa, Uganda in East Africa and Botswana and South Africa in Southern Africa. The aim of this review was to provide a quantified estimate of human resources for health in these five countries. In each of these, we also aimed to estimate the proportion of health workers currently working in primary health care, to compare this to estimates of numbers needed and to assess how the situation has changed in recent years.

## Methods

Data were collected both through a formal literature search of on-line databases (WHO Global Health Observatory [7] and World Health Statistics [8]) and by in-country searches for unpublished “grey” literature, including informal surveys and formal reports by government and non-governmental agencies at local and national levels. For each country, we conducted a literature search in online databases (Embase, MEDLINE, Global Health, CINAHL) using terms for primary health care (community/rural/family/primary healthcare/care/service/centre; general practice; primary health care; community health services; community health nursing; rural health services; community health centres) and terms for health workers (doctor/physician/nurse/midwife/traditional birth attendant/traditional healer/village health worker). No language restrictions were applied. We made a particular effort to find “grey” literature and unpublished statistics by contacting key stakeholders in ministries of health, universities and non-governmental organizations (NGOs) in all participating countries. We requested data on numbers of health workers in post and estimates of numbers needed, with a focus on primary health care structures (as defined by the ministry of health in each country). Data were selected for inclusion by authors from each of the included countries, and these were coordinated by one of the authors (MLW) in order to standardize the results as far as possible. Health worker densities were calculated using the numbers of health workers from the sources above and official statistics on the country’s population [7]. Most of these unpublished sources were not explicit about their methods, so it is not possible to evaluate their reliability.

## Results

The demographic characteristics and child/maternal health statistics of the five chosen countries are summarized in Table 1. There is debate about the accuracy of published estimates of the maternal mortality rate in particular, so data for this indicator are included from two sources applying different standard methodologies (the estimates for Botswana from these sources differ by 2.5-fold) [9,10].

**Table 1:** **Comparison of indicators for the selected countries**

	**Uganda**	**Mali**	**Sudan**	**Botswana**	**South Africa**	**Source**
Population in 2010 (millions)	33.4	15.4	33.0	2.0	50.1	[30]
Annual population growth (2000–2010)	3.2%	3.1%	2.4%	1.3%	1.1%	[30]
Total fertility rate (per woman) in 2010	6.1	6.3	4.4	2.8	2.5	[7]
Maternal mortality rate 2013 (per 100 000 live births)	360	550	360	170	140	[10]
	324.9	388.3	275.2	480.8	174.1	[9]
Stillbirth rate 2009 (per 1000 total births)	25	23	24	16	20	[30]
Proportion of births attended by skilled personnel, % (year of latest data available)	58.0 (2011)	58.2 (2011)	19.9 (2010)	99.1 (2010)	91 (2003)	[7,23]
Neonatal mortality rate 2010 (per 1000 live births)	26	48	35	19	18	[30]
Under 5 mortality 2010 (per 1000 live births)	99	178	103	48	57	[30]
Government health expenditure as % of GDP (2009)	8.5	5.5	7.3	10.0	9.2	[30]
Government health expenditure (PPP Int $ per capita) (2009)	26	27	44	985	407	[30]

### Overall density of doctors, nurses and midwives

**Figure 1:**
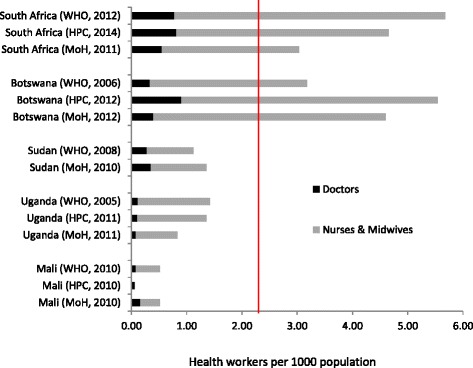
**Health workers per 1000 population in five African countries, according to different sources (** ***red line*** **indicates the level below which countries are said to have a “critical shortage” of health workers, according to WHO).** MoH: ministry of health statistics [11,20,37-39]; HPC: health professionals’ councils [11,29,37,38]; WHO: most recent available data from [7].

Figure 1 shows the numbers of doctors, nurses and midwives per 1000 population from different sources (ministry of health, professional councils and WHO). Through the WHO global health observatory, the latest available statistics date from 2005 for Uganda, 2006 for Botswana and 2008 for Sudan. Only Mali was able to provide comprehensive figures which included workers in the private sector and in community health centres [11]. Irrespective of the source of data, it is clear that Mali, Uganda and Sudan all fall short of WHO’s recommended target of 2.28 health workers per 1000 population, whereas South Africa and Botswana both exceed it.

**Figure 2:**
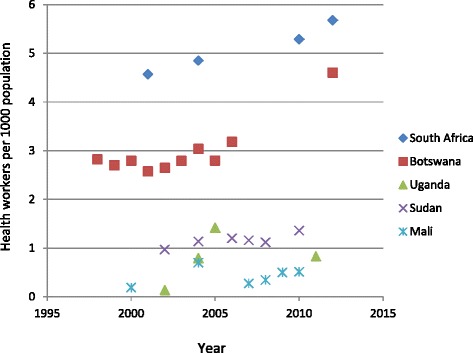
**Time trends in health worker density per 1000 population in 5 African countries.** (Data from WHO [7,8] except for the 2010–2012 figures for Botswana, Sudan and Uganda which are taken from national ministry of health statistics [37-39]).

Health worker density has been increasing steadily in wealthier countries such as South Africa and Botswana. However, after an initial increase from 2000-2004, there has been no further increase since 2004 in lower income countries such as Sudan, Mali and Uganda (Figure 2).

### Training

**Figure 3:**
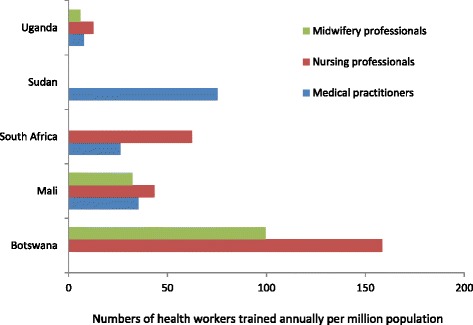
**Numbers of health workers trained annually (within each country) per million population.** (NB: in South Africa, numbers of nurses and midwives are combined; no data was available from Sudan on numbers of nurses and midwives trained.) *Sources*: [39,40]; Jacques Appelgryn, personal communication; HEMIS database, South Africa; Sidibé, personal communication; Register of graduations, Faculty of Medicine, Pharmacy and Dentistry, University of Bamako; [41].

Training of health workers has been seen as key to solving the human resource crisis in Africa, but the nature and impact of recent training initiatives on health worker density has been mixed (see Figure 3). Countries have taken very different approaches to training. Botswana trains relatively large numbers of nurses and midwives, but until recently, there has been no medical school so Batswana doctors could only be trained abroad (most of whom never returned). The majority of doctors in Botswana have migrated from other African countries [12]. South Africa has eight medical schools, but the majority of doctors work in secondary care and many in private practice. Sudan trains large numbers of doctors but relatively few nurses and midwives (data on nurses/midwives trained were not available). It is estimated that over 60% of Sudanese physicians practice outside the country. Every year, over 3000 doctors graduate in Sudan, but around 800 of these emigrate. Non-medical health professionals are also emigrating but to a lesser degree [13]. Mali trains more doctors per capita than South Africa or Uganda, but every year, there are posts in government health services for only about one quarter of the newly qualified doctors. There has been no quantitative survey of new doctors in Mali to track the numbers of doctors who leave, but qualitative interviews with key stakeholders report that most work in private clinics within Mali, some are employed as representatives for the pharmaceutical industry and some migrate abroad. In Uganda, a survey of alumni from Mbarara University of Science and Technology, which was set up in 1989 to train doctors for community health work, has shown that only 35% of its 790 medical graduates are currently working for the government, while 51% are working for HIV/AIDS-related NGOs and 12% have left the country [14].

### Primary health care staffing levels

The structure of primary health care providers and services, as defined by the ministry of health in each country, is summarized in Table 2. Although the details differ, the types of primary care providers are similar in all the countries. Specific figures on staffing of primary care facilities were found for four of the five countries (Botswana, Sudan, Mali and Uganda), although we could not find figures on staffing of broader primary health care (beyond health facilities). Some of the primary care facilities described provide services which may be regarded as a secondary care function in other countries (for example, small inpatient unit, maternity unit). The table does not include primary care services provided by secondary care facilities.

**Table 2:** **Description of primary health care providers and services in five African countries**

**Level**	**Details**	**Uganda**	**Mali**	**Sudan**	**Botswana**	**South Africa**
Traditional health practitioners (THPs) and traditional birth attendants (TBAs)	Personnel	Many THPs practice although there is no national association. TBAs are banned from conducting deliveries.	5875 THPs are registered with 135 local associations, and there is a national federation of THPs’ associations. TBAs conduct deliveries.	Traditional healers (TH) are practising all over Sudan. Also, there are many TH centres that belong to special religious groups (Tarriga). TBAs (Habil Midwives) are doing home deliveries.	3100 THPs are registered with their associations	About 185 500 traditional African healers. 29 000 belong to traditional healer organizations.
Community health workers	Name	Village health teams (VHTs)	“Relais” and “Agents de Santé Communautaire” (ASCs)	Community health workers (CHWs); mother support groups.	Village health committees, led by nurses from the primary clinic	Community health workers
	Personnel	Volunteer community members	Volunteer community members	CHWs are paid community members, active and motivated to help in providing care	920 community home-based volunteers and in some places community health nurses	80 000 – young (mostly matriculants) with basic and non-standardized training of 10 days to 1 year.
	Roles	Health promotion. Integrated Community Case Management (ICCM) is being piloted.	Relais: mobilizing villagers for vaccination. ASCs: screening for malnutrition; ICCM	Help in providing essential PHC services addressing community needs	Provide basic care to patients with terminal or debilitating conditions in the home setting, under supervision of registered nurses	Mostly health screening and education, follow up on adherence and social problems
Lowest level health facility	Name	Health centre II	Maternité	Basic health unit (BHU)	Mobile stops (outreach clinics), health posts	Primary health care clinic (occasionally mobile clinic)
	Personnel	9 staff, led by enrolled nurse or midwife	Matrone (midwifery assistant)	Medical assistant/nurse/midwife	Registered nurses	5–23 nurses (professional and enrolled)
	Services	Basic curative consultations; preventive interventions; emergency deliveries	Basic curative consultations; antenatal care and normal deliveries	Basic comprehensive services, MCH	Basic preventive (immunizations) and curative services	Basic preventive (immunizations) and curative services per standard treatment guidelines for nurse management
	Population covered	5000	Not specified	5000	400–500	10 000
Next-level health facility	Name	Health centre III	Centre de Santé Communautaire (CSCOM)	Family health centre (FHC)	Primary care clinics with and without maternity	Community health centre (with and without maternity obstetric units (MOUs))
	Personnel	19 staff, led by a generalist doctor; most are led by a nurse or midwife	Usually led by a nurse; few have a doctor.	Planned to be led by family medicine physician/GP or medical officer	Registered nurses and midwives	1–5 medical officers, 30–92 nurses and midwives
	Services	As above, plus laboratory services, maternity and small inpatient unit	As above, plus responsible for vaccination, etc. in health subdistrict	Comprehensive services including MCH/non-communicable diseases.	Preventive and curative services and antenatal and postnatal services.	Comprehensive services, usually including deliveries
	Population covered	20 000	15-km radius, c. 20–30 000	20 000	1000–3000	50 000

#### Mali

**Figure 4:**
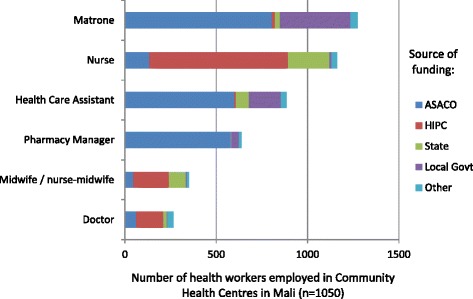
**Numbers of health workers in the 1050 community health centres in Mali, according to source of funding.** ASACO: Association de Santé Communautaire (Community Health Association, funded by user fees); HIPC: Heavily Indebted Poor Countries fund (money given to the state through debt cancellation). *Source*: [42].

Detailed figures on staffing of community health centres were only available for Mali (Figure 4). These are financed largely by user fees, managed by a community health association (Association de Santé COmmunautaire or ASACO). The ASACOs report their staffing data to the National Federation of ASACOs (FENASCOM), who publish this data. In the majority of cases, user fees are only used to employ lower level workers such as a matrone (midwifery assistant), a health care assistant or a pharmacy manager. Nurses were the only skilled workers in the majority of community health centres; few have a qualified midwife or doctor. The majority of skilled workers were funded by the state, either directly or using money from debt cancellation (Heavily Indebted Poor Countries or HIPC fund). Consequently, only 10.2% of doctors and 40.4% of nurses and midwives work in community health centres [15].

#### Uganda

**Figure 5:**
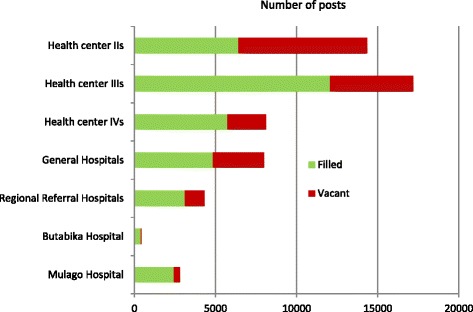
**Numbers of posts vacant and numbers filled at different levels of the health system in Uganda.** *Source*: [17,43].

In Uganda, data are available on overall staffing levels at health facilities, compared to posts available (Figure 5). Although these include both clinical and non-clinical posts, there is a much higher proportion (and absolute number) of vacancies in primary care facilities (health centre IIs and IIIs) compared to secondary care facilities (health centre IVs and general hospitals). Vacancies are calculated in relation to staffing “norms” which have not changed since 2005. A vacant post is defined as a post which exists in the plan for the health facility but where no person has been recruited to work in that role. Unfortunately, the published statistics do not differentiate between clinical and non-clinical vacancies. Even if all these posts were filled, this would be less than the level of health workers recommended by WHO.

It is very clear that both the absolute number and proportion of vacancies are much higher at the lowest levels of the health care system (Figure 5). There has been some improvement in staffing levels thanks to a large recruitment drive in 2012–13, particularly aimed at health centre IIIs and IVs. The percentage of posts filled at these facilities increased from 46% to 70% and from 55% to 71%, respectively, from 2009 to 2013 [16,17], and there was a small decrease in posts filled in general hospitals. However, at the lowest level of primary health care (health centre IIs), there was a much smaller increase, from 36% to 45% of posts being filled.

#### Botswana

Botswana has data on overall posts and vacancies in primary health care facilities. Overall, only 34% of health workers are employed to work in primary health care facilities; only 5% of posts are currently vacant, but a substantial increase has been recommended for 2016 (from 2209 to 4722), both in primary and secondary care [12]. By 2016, it is projected that 38% of posts will be in primary health care, and 53% are expected to be vacant [12].

#### Sudan

Two thirds (67%) of the available health workers are employed in secondary and tertiary health care while only 33% work in primary health care services [18]. There are no data specifying exactly how the health workers are distributed in primary or secondary health care facilities.

#### South Africa

At the national level, data are available on numbers and targets of family physicians. The national Human Resources for Health policy aims for 0.2 family physicians per 10 000 population, which equates to 1060 for the whole of South Africa. There are currently 545 family physicians on the new register [19], and in 2008, 790 family physicians were working in South Africa, 521 in the public sector and 269 in the private sector [20]. However, most primary health care services in South Africa are not delivered by family physicians.

Comprehensive information on staffing of primary health care facilities was not available at the national level but was available from a survey of 340 primary health care facilities in six of the poorest districts across four provinces of South Africa [21]. The Workforce Indicators of Staffing Need (WISN) method was used to calculate the required human resources. Overall, the number of doctors in primary health care was only 7% of the required number. Many clinics did not receive visits from doctors, and community health centres had too few doctors. Some districts had no doctors at all for primary health care. While the total number of professional nurses was 94% of the target, two districts had overall excesses and four had overall shortages. There was uneven distribution, with some clinics having too many nurses while others had a shortage. The numbers of enrolled nurses and enrolled nurse assistants were 60% and 83%, respectively, of what was needed.

### Skilled birth attendants

**Fig. 6:**
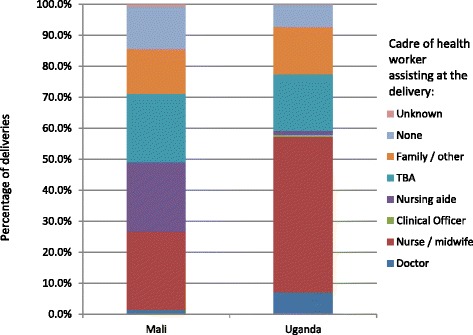
**Cadre of health worker assisting at deliveries in Mali and Uganda [** 22 **,** 23 **].**

The percentage of deliveries assisted by a “skilled” birth attendant (SBA, which WHO defines as a doctor, nurse or midwife – see Table 1) is one of the few indicators of primary care staffing available for all countries. Nurses and midwives form the majority of “skilled” birth attendants but still only assist at a minority of deliveries in lower income countries such as Mali (Figure 6), where 22% of deliveries are assisted by a “matrone” (a midwifery aide with 6 months training, considered “skilled”). In both Mali and Uganda, 18-22% of deliveries are assisted by traditional birth attendants (who are not considered “skilled”). Twenty-eight per cent of births in Mali and 22% in Uganda are not assisted at all or only by family members. These statistics mask significant economic and geographic inequalities: the rate of skilled birth assistance more than doubles from the poorest to the wealthiest quintile. In the poorest half of the population, over half of deliveries are assisted by traditional birth attendants (TBAs), family members or no one at all [22,23].

### Lay and traditional health workers

Statistics on lower level health workers are not widely available. Numbers of community health workers were only available for Botswana (920, that is, 0.48 per 1000) and South Africa (80 000, that is, 1.58 per 1000). Mali, Botswana and South Africa have associations of traditional healers, who have statistics on their membership see Table 2; however, members probably represent a minority of all traditional primary care providers (traditional healers and traditional birth attendants).

## Discussion

### Principal findings

Analogous to the “inverse care law” reported by Tudor Hart in 1971 [24], there is an “inverse primary care law” operating within the five African countries surveyed, which is that staffing (especially in primary care settings) is inversely related to poverty and level of need [25]. Staff shortages are greatest in primary care and in the lowest level health facilities and in the poorest areas within the lowest income countries. There is an intention to provide primary care services outside the hospital in all the countries surveyed. For instance, in Uganda, there are many buildings for health centre IIs and IIIs. The problem is that they are not adequately staffed and often do not provide comprehensive primary care services. Health worker density is not increasing in the lowest income countries because population growth is clearly outstripping growth in numbers of health workers. This situation is common to many African countries [26].

Although training initially helped to improve health worker density, other measures are also needed to attain targets in low-income countries. Currently, the majority of doctors trained in Mali, Uganda and Sudan do not stay to work in government health services in their countries, let alone in primary health care, because insufficient resources are spent on recruiting or retaining them, and they would face a poor working environment, difficult living experiences and a poor career path [25]. Health workers migrate to better paid jobs, either within their country or abroad [27]. Wealthier countries within Africa employ health workers from lower income countries.

In spite of calls for better data on human resources for health since the World Health Report in 2006 [6], data on the health workforce are still imprecise and insufficient especially as regards primary health care settings and lower level health workers.

### Study limitations

This study was limited to the countries participating in the HURAPRIM project which may not be representative of other African countries. It is also important to recognize that primary health care function can be provided by health facilities (such as emergency departments and hospital outpatient departments) which are not formally recognized as part of primary care. At the same time, we had no formal measure of the extent to which even the primary care facilities were offering holistic patient-centred care as envisaged in the Alma-Ata declaration.

The reliability of the data could not be evaluated, so results of the analysis should be treated with care. There are still gaps and discrepancies in the available data, because information systems in low-income countries are weak. In several countries, ministry of health figures are an underestimate because they do not all include health workers in the private sector. In South Africa, the ministry of health statistics include doctors and nurses in both the public and private sectors [20], but the numbers are much lower than those in professional registers [28,29]. One explanation for this is that many South African doctors and nurses are still registered in South Africa but are working abroad or in non-clinical roles in South Africa. The numbers are lower than those given by WHO, which also include unqualified nursing auxiliaries. In Uganda, the health professionals’ councils’ statistics overestimate the number of health workers because their registers are not always regularly updated and may include workers who are no longer currently working in the country. In Mali, not all current health workers seem to be registered with the health professionals’ councils. Sudan was divided into two countries in 2011, which also makes interpretation of data more difficult. As of August 2015, the WHO Global Health Observatory does not have data on health worker density after 2005 for Uganda, after 2009 for Botswana or after 2008 for Sudan.

It is not explicit from which sources WHO [30] obtained its figures, but it seems that the sources may not always be consistent. For example, the steep rise in health workers from 2004 to 2005 in Uganda can only be explained by the data in 2004 coming from ministry of health statistics, whereas the data for 2005 is consistent with data from the health professionals’ councils. The WHO figures for nurses and midwives in South Africa [7] are identical to the total number of nurses, midwives and nursing auxiliaries registered with the South African Nursing Council [29] – which is misleading because it includes a large number of “enrolled nursing auxiliaries” who have only 1 year’s training and are not qualified nurses or midwives.

Detailed data on health workers in primary care were only available in Mali (collected by the national federation of community health organizations), and only Uganda and Botswana attempted to calculate the per cent of “vacancies” at different levels of the health system. Even data on SBAs are not completely comparable, because the definition of SBA varies from country to country. In Mali, “SBAs” include “matrones” (midwifery assistants who have had 6 months of training) whereas in Sudan “SBAs” do not include village midwives (who have had 2 years of training). This may explain the large disparity between Mali and Sudan in the percentage of births attended by a SBA. Furthermore, the WHO definition does not include certain cadres of health worker who may be skilled in delivery (such as “clinical officers”, “medical assistants” or even trained TBAs). Estimates of maternal mortality are also imprecise and vary widely according to different sources (Table 1, [9,10]), which makes it difficult to measure impact of policies on key public health goals such as reduction of maternal mortality.

### Policy implications

Better data are needed on human resources for health, in particular in primary health care. WHO has proposed indicators and methods for monitoring human resources for health, but these do not include any indicators specifically about primary health care [31]. We recommend that future reports on human resources from WHO, ministries of health and other stakeholders describe clearly how the data was collected. This information could then be used to create a classification of data sources, from more rigorous to doubtful, in order to assess the quality of the data.

Whichever method is used to calculate the numbers of health workers needed, it is clear that targets are unattainable in many low-income countries in the foreseeable future [26]. Existing resources are insufficient to train and employ enough staff to deliver high-quality primary health care in many African countries, so there is a need to lobby for increased funding. Several donor-funded programmes such as MEPI are spending substantial resources on training health workers [32], but their impact will be limited unless resources are also available to recruit and retain these health workers within the government health system. Currently, most physicians in the included African countries do not stay to work in public health services, let alone in primary health care. If there were appropriate measures to recruit and retain doctors in primary health care, they could contribute to improving the quality of care through supervision of teams [33].

Until sufficient trained health workers are available, it is also essential to use existing staff resources more effectively, improving their distribution and productivity, and to prioritize initiatives to facilitate the efficient working of, and reduce demand for, the scarce resource of health workers in post. Such initiatives include training non-professional support staff and village health workers. As funds are always limited, it is very important to ensure that they are used in the most cost-effective manner. This requires a more considered and balanced approach to building up the primary health care team with optimal staff and skill mix appropriate to local realities and enabling Africans to achieve quality health care and public health gains. Rather than focussing only on numbers of health workers needed, it may be more realistic in the short term to consider how, within available resources, effective primary care interventions can be delivered in order to achieve key public health targets such as reductions in maternal and child mortality, as well as effective management of chronic diseases.

In the short term, public health impact may be greater if there were a better balance between spending on training physicians, spending on recruitment and retention of physicians in public health services and spending on improving the quantity and quality of training and supervision for nurses, midwives, middle- and low-level health workers. For example, nursing/midwifery assistants and TBAs are in practice attending the largest proportion of deliveries for the poorest people in the lowest income countries. There is no reason why such cadres could not be trained to improve the quality of essential primary care. Indeed, there is good evidence that training traditional birth attendants to follow good practice, particularly in onward referral, can reduce perinatal mortality rates [34,35].

### Priorities for further research

As shown in the few countries with detailed data, overall staffing numbers mask underlying inequalities between rich and poor, urban and rural and secondary and primary health care. It may be useful to have more accurate statistics on health workers available versus the numbers needed in primary health care, to enable more efficient planning and deployment of human resources. However, existing methods for estimating human resource needs all have their limitations, and new methods need to be developed specifically for primary care [36].

Although data collection is necessary, it is not sufficient: the process must be designed to influence policy. In Uganda, the Capacity Project and ministry of health have spent several years building up a human resource information system in Uganda. This may have influenced the increased recruitment of health workers which took place in 2013, but there still remain a large number of vacancies, particularly in the lowest level of primary care facilities [17]. An urgent priority for research is to find ways of collecting and presenting data which are effective in influencing policy on human resources for health.

It is also important to research how to maximize the impact of health workers on important public health targets, such as reducing maternal and child mortality and better managing chronic diseases. Paradoxically, the only two countries in our sample which have achieved WHO’s threshold of health worker density (Botswana and South Africa) both experienced increased maternal mortality from 1990 to 2003, probably because of the HIV/AIDS epidemic. Maternal mortality reduced again from 2003 to 2013, probably due to increased number of HIV-positive pregnant women receiving adequate and early treatment, but still has not returned to 1990 levels [9].

## Conclusions

The “inverse primary health care law” is a reality in the five African countries included in this study. The focus on attaining a certain health worker density has encouraged governments and funders to focus on training more doctors, nurses and midwives, but this has not resulted in improved recruitment and retention in primary care. The number of health workers per unit population has not increased in the lowest income countries, and consequently, those with the greatest need have the worst access to effective services.

Additional resources are needed, but it may also be possible to use existing resources more cost-effectively. This depends on better leadership, with an emphasis on recruitment and retention of skilled staff in training and supervisory roles, to support the lower level health workers who currently provide the front line of primary health care in most of Africa. Such improvements in the management and quality of the health workforce are essential for delivering effective life-saving interventions in primary health care and achieving key public health goals such as the reduction of maternal and child mortality. Information systems need to be improved in a way that informs policy on the health workforce, because at present there is a serious problem with the quality of data, especially on staffing of primary care services.
